# Safety and Efficacy of Ab Interno XEN 45 Gel Stent in Patients with Glaucoma and High Myopia

**DOI:** 10.3390/jcm12072477

**Published:** 2023-03-24

**Authors:** Matteo Sacchi, Antonio M. Fea, Gianluca Monsellato, Elena Tagliabue, Edoardo Villani, Stefano Ranno, Paolo Nucci

**Affiliations:** 1 University Eye Clinic, San Giuseppe Hospital, IRCCS Multimedica, University of Milan, 20123 Milan, Italy; 2 Department of Ophthalmology, University of Turin, 10124 Turin, Italy; 3 MultiMedica IRCCS, 20099 Milan, Italy; 4 Department of Clinical Sciences and Community Health, University of Milan, 20122 Milan, Italy; 5 Ophthalmology Department, Circolo and Fondazione Macchi Hospital, ASST Sette Laghi, 21100 Varese, Italy; 6 Department of Biomedical, Surgical and Dental Sciences, University of Milan, 20122 Milan, Italy

**Keywords:** glaucoma, glaucoma surgery, stent gel implant, Xen, trabeculectomy, hypotony, hypotony maculopathy

## Abstract

This study reports on the safety and efficacy of Xen 45 in patients with glaucoma and high myopia. It was a retrospective study including patients with high myopia (>6D) who underwent Xen implant with 2 years of follow-up. The primary outcome was to report the incidence of hypotony (IOP ≤ 5 mmHg) and hypotony-related complications. Patients with high myopia treated with mitomycin-C-augmented trabeculectomy were included as a control group. We included 14 consecutive patients who underwent Xen implant (seven eyes) and trabeculectomy (seven eyes). The mean myopia was −14.71 ± 5.36 and −15.07 ± 6.11 in the trabeculectomy and Xen groups, respectively (*p* > 0.05). The success rate and the mean IOP at 1 and 2 years from the intervention were statistically comparable between the two groups. The group undergoing trabeculectomy showed a higher incidence of hypotony (six eyes (85.71%) vs. two eyes (28.57%)) and hypotony maculopathy (three eyes (42.86%) vs. zero eyes (0%)) and required more postoperative procedures. Patients with high myopia were at higher risk of hypotony-related complications after trabeculectomy. The Xen implant can achieve an IOP control comparable to trabeculectomy with a significantly better safety profile and can be considered as an option for the management of patients with high myopia and glaucoma.

## 1. Introduction

The only proven treatment for the management of glaucoma is to decrease intraocular pressure (IOP). In the glaucoma treatment algorithm, the surgical approach is considered after the failure of medication and laser. Indications for surgery are uncontrolled IOP, progressing disease despite apparent IOP control, and intolerance to active agents. Although several surgical options are available, and mini-invasive glaucoma surgeries are rising in popularity, trabeculectomy is still the most performed glaucoma surgical technique [1,2,3].

Although trabeculectomy can effectively lower IOP, significant complications can occur postoperatively, including hypotony [4].

The term hypotony refers to an IOP value of 5 mmHg or less [5].

In clinical practice, hypotony occurring within 3 months after surgery is defined as “early,” whereas hypotony still persistent 3 months after surgery is defined as “chronic” [6,7,8].

Hypotony is one of the most common complications [9], potentially leading to clinically significant and sigh-threatening conditions such as choroidal effusion, optic nerve edema, and maculopathy [5]. Since the 1990s, myopia has been associated with the development of hypotony maculopathy after filtering surgery [10,11,12], and a large retrospective study confirmed that myopia was significantly associated with hypotony maculopathy [5].

In order to improve the predictability and the safety profile of glaucoma surgery, several minimally invasive glaucoma surgical (MIGS) techniques have been studied and developed in the last years and are nowadays available [13,14].

The Xen 45 gelatin stent (Allergan, Dublin, Ireland) is a 6 mm device implant with a 45 μm internal lumen designed to create a conjunctival filtering bleb [15].

The advantages of this less invasive, bleb-forming technique are a more controlled aqueous humor outflow; less tissue manipulation; quick recovery; and a lower incidence of complication, including hypotony, compared to trabeculectomy [16,17].

In this retrospective study, we report the efficacy and safety of Xen 45 in consecutive patients with uncontrolled glaucoma and concomitant high myopia (>6D). We included in the analysis consecutive patients with glaucoma and high myopia who underwent trabeculectomy as a control group.

## 2. Methods

We conducted a retrospective medical chart review using the hospital’s electronic database (e4cure, Exprivia, Molfetta, BA, Italy). The search period was between 1 January 2016, and 1 January 2020, and patients referred to the glaucoma service were screened.

Consecutive patients with myopia >6 D, 18 years or older, with available data at the established post-operative time points and with a follow-up of at least two years who underwent filtration surgery (Xen implant or trabeculectomy) between 1 January 2016, and 1 January 2020, were included in the study. All the surgeries had to be performed by the same glaucoma-trained surgeon (MS), and both standalone and combined surgery with phacoemulsification were considered eligible for the analysis. Patients who underwent laser trabeculoplasty at least six months before the surgery were also considered eligible.

Patients underwent surgery according to the clinical judgment when meeting one of the following criteria: (a) unmet target intraocular pressure (IOP) despite maximum tolerated medical therapy (including oral acetazolamide) and laser; (b) a significant perimetric progression confirmed on three consecutive reliable visual fields (VF) (Humphrey field analyzer II 750-Carl Zeiss Meditec Inc.; Dublin, CA-30-2 test, full-threshold); (c) intolerance to medical therapy.

Exclusion criteria were previous surgery, except for cataract surgery, if performed at least 6 months before the Xen implant; anterior chamber intraocular lens; neovascular, uveitic, angle-closure, and syndromic glaucoma.

We used the threshold of >6D for the definition of high myopia [18], and the threshold of ≤5 mmHg for the definition of hypotony [5].

For our analysis, the following demographic and clinical data were collected: age, gender, diabetes status, refraction, axial length, best-corrected visual acuity (BCVA), lens status, type of glaucoma, baseline IOP, corneal thickness, number of glaucoma medications, VF mean deviation.

The primary outcome of the analysis was to report the incidence of hypotony and hypotony-related complications, including maculopathy in patients with glaucoma and high myopia after Xen 45 ab interno implant and after trabeculectomy. Secondary outcomes were to report other major and minor complications, assess the IOP lowering efficacy, and compare the safety profile and the efficacy of Xen 45 with trabeculectomy.

The success of the procedures was defined as an IOP between 5 and 15 mmHg at any follow-up visit. As stated by the World Glaucoma Association consensus group, the success was complete if without medicines and qualified if with drugs [19].

Patients undergoing other IOP lowering procedures were considered failures.

The study was carried out at the Ophthalmic Clinic, San Giuseppe Hospital, IRCCS Multimedica, University of Milan. This study was approved by the Institutional Review Board (San Giuseppe Hospital, Milan—IRCCS Multimedica) and adhered to the tenets of the Declaration of Helsinki. We informed the patients about processing personal data, and we obtained consent from all the patients before their data were part of this analysis.

### 2.1. Surgical Technique

#### 2.1.1. Trabeculectomy

The trabeculectomy technique has been previously described [20].

Briefly, trabeculectomy was performed under a peribulbar block. After a traction suture was passed through the cornea, a fornix-based conjunctival flap was dissected, and a partial-thickness scleral flap was fashioned. A total of 0.3 mg/mL mitomycin (MMC) was applied with sponges left for 2.5 min under the Tenon-conjunctival layer.

After MMC was carefully washed-out with a balanced salt ophthalmic solution, a punch was used to perform sclerotomy, and peripheral iridectomy was performed in each patient. The scleral flap was sutured with two-four 10-0 nylon sutures, and the conjunctiva and Tenon’s layer were closed with polyglactin sutures. A 10-0 nylon suture was used to close the Tenon-conjunctival layers to the cornea.

#### 2.1.2. Xen Implant

Xen surgeries were performed under topical anesthesia. After 0.1 mL of mitomycin-C (MMC), 0.2 mg/mL was injected within the upper bulbar conjunctiva, the main and the side port corneal incisions were made, and the anterior chamber was filled with viscoelastic (Healon GV® PRO, Johnson & Johnson Surgical Vision, Inc. 1700 E. St. Andrew Place Santa Ana, CA 92705 USA 1-877-266-4543); the Xen was placed in the superior nasal quadrant. The surgeon’s goal was to obtain the Xen gel implant to be positioned following the “3:2:1 rule”: 3 mm under the conjunctiva, 2 mm intrascleral, and 1 mm left in the anterior chamber. At the end of the surgery, the proper placement of the Xen in the anterior chamber was verified using a goniolens and viscoelastic washed-out.

Patients were instructed to withdraw topical and systemic ocular hypotensive medications on the day of surgery. Postoperative therapy for both groups consisted of a topical steroid/antibiotic combination for the first two weeks, then switched to a topical steroid, tapered in 4–6 months. Cyclopentolate was prescribed after trabeculectomy twice a day for the first two weeks and then according to the physician’s judgment.

### 2.2. Statistical Analysis

Data were summarized as mean ± standard deviation, if continuous, or frequencies and percentages, if categorical. Differences between trabeculectomy and Xen were evaluated for each variable by the non-parametric Wilcox test, if continuous, or Fisher’s exact test, if categorical. As efficacy outcomes, IOP values were collected at the following time points after surgery: 1 day, 1 week, 1 month, 3 months, 6 months, 12 months, and 24 months. Values were then compared between groups by the non-parametric Wilcoxon test and longitudinally by mixed models. Kaplan–Meier curves were drawn to graphically represent differences between groups in the probability of success, defined as IOP values between 5 and 15 mmHg, and the log-Rank test was used to evaluate the differences between the curves.

## 3. Results

In total, 18 eyes of 18 patients were identified: 9 eyes received a Xen, and 9 underwent trabeculectomy. We excluded two patients per group for insufficient follow-up, one patient in the trabeculectomy group for previous glaucoma surgery, and one patient in the Xen group for diagnosis of uveitic glaucoma.

A total of 14 eyes, 7 in the Xen and 7 in the trabeculectomy group, were examined. The follow-up was 2 years. The mean age and mean deviation in the Xen group were lower compared to the trabeculectomy group (52.96 ± 10.15 vs. 63.99 ± 13.06, and −10.88 ± 5.59 vs. −16.17 ± 5.68 in the Xen and trabeculectomy groups, respectively); however, these differences were not statistically significant. All patients met the diagnostic criteria of primary open-angle glaucoma. Other baseline characteristics were comparable (Table 1).

One patient per group received simultaneous glaucoma and cataract surgery. Eyes treated with trabeculectomy had a lower IOP throughout the follow-up than those treated with Xen. The difference was statistically significant from 1 week to 6 months after surgery in favor of trabeculectomy, while it was comparable after 1 and 2 years from intervention (10.00 ± 2.83 vs. 12.00 ± 1.90, p 0.22, and 12.83 ± 4.62 vs. 14.83 ±3.97, p 0.8717, trabeculectomy vs. Xen at 1 and 2 years, respectively; Table 2).

**Figure 1:**
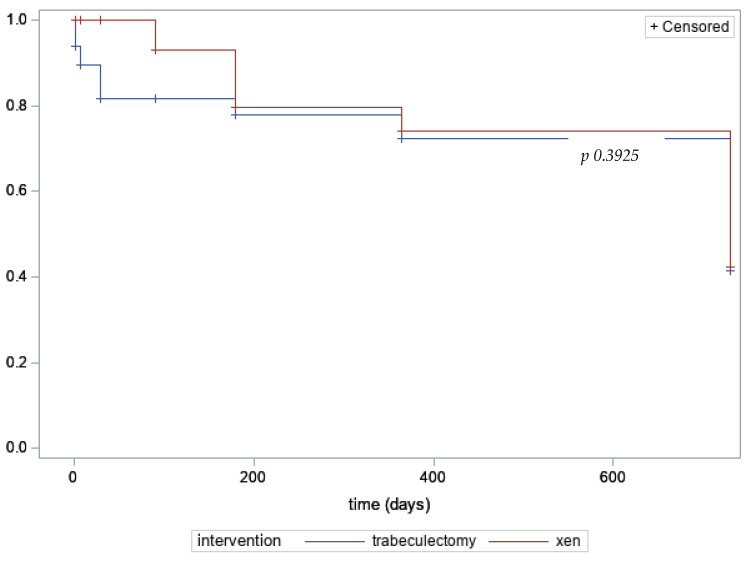
Kaplan–Meier curves for success of estimated probability (%).

Xen and trabeculectomy showed an equal probability of success at 2 years of follow-up (41.31% ± 1.96 vs. 42.23% ± 1.96, p 0.3925, log-Rank test; Figure 1).

At the last follow-up, and starting from 1 year after surgery, one patient per group was using a prostaglandin analogue, and one patient per group was under a fixed combination of timolol and dorzolamide. The number of hypotony and hypotony-related complications was significantly higher in the trabeculectomy group. Hypotony maculopathy was recorded in 42.86% of eyes after trabeculectomy, whereas none of the eyes in the Xen group experienced this complication (Table 3). All the cases of hypotony maculopathy occurred in the first month after surgery.

Patients in the trabeculectomy group required more post-operative procedures (Table 4).

Patients requiring bleb needlings and surgical bleb revision were similar in the two groups, but more myopic patients undergoing trabeculectomy required intrableb autologous blood injection and AC refilling with viscoelastic compared to patients who underwent Xen implant (42.86% vs. 0% and 28.57% vs. 0% in trabeculectomy and Xen groups, respectively; Table 4).

## 4. Discussion

To the best of our knowledge, this is the first study reporting a dataset of the safety and efficacy of ab interno Xen 45 and trabeculectomy in a subset of patients with high myopia. Recently, we published a retrospective, multicenter study on Xen implant in patients with high myopia [21]. In the current study, we introduced as a control group consecutive patients with uncontrolled glaucoma and high myopia (>6D) who underwent trabeculectomy.

The primary outcome of our analysis was to report the incidence of hypotony and hypotony-related complications.

Overall, throughout 2 years of follow-up, Xen 45 implant showed a lower incidence of complications compared to trabeculectomy. None of the patients with glaucoma and high myopia treated with ab interno Xen 45 implant experienced hypotony maculopathy, in contrast to what we observed in the trabeculectomy group.

The incidence of hypotony after trabeculectomy varies greatly in the literature, and it has been reported in up to 42% of patients. In a retrospective, 5-year study involving 123 patients, late hypotony occurred in 42.2% of eyes, hypotony maculopathy in 8.9%, and 14.9% of eyes had a significant loss of vision (four lines of visual acuity) [22]. A large retrospective study involving nine centers in the UK and 395 patients followed up for at least 2 years reported the presence of late hypotony (>6 months) in 7.2% of cases. Patients who experienced hypotony had a significantly higher risk of losing vision than the whole group (10% vs. 6%) [4]. The TVT study, a large RCT comparing the safety and efficacy of tubes with trabeculectomy, showed similar results, reporting persistent hypotony (>3 months) in 12.3% of patients who underwent trabeculectomy [6]. Different exposure times, concentrations, and areas of application of MMC and the use of different sutures (adjustable, releasable) can at least partially explain the wide range of hypotony reported in the literature [4].

Myopia has been recognized as a risk factor for hypotony after glaucoma surgery since the 1990s [10,11,12], and more recently, large retrospective studies [5] and reports [23,24] confirmed the link between the onset of hypotony [5] and hypotony maculopathy [23,24] after trabeculectomy in patients with glaucoma and myopia.

As described by Gass, hypotony maculopathy is caused by the sclera bending inward, associated with retinal and choroidal folds over the posterior pole [25]. Both levels of IOP and biomechanical properties of the sclera are considered crucial in the development of hypotony maculopathy. Myopic eyes have a thinner sclera, particularly at the posterior pole, due to the loss of extracellular matrix and the reduction in collagen fibril diameter [26]. These biomechanical changes lead to an overall reduced scleral stiffness in myopic eyes [27]. Due to the lower thickness and rigidity, the myopic eyeball is more likely to collapse, making myopic patients at higher risk of hypotony maculopathy in cases of hypotony after filtering surgery [28]. Published studies reported a rate of hypotony maculopathy up to 20% after trabeculectomy [29,30]. In our work, we found a rate of 42.86% of hypotony maculopathy after trabeculectomy in a high-risk population of highly myopic eyes. The populations and the methods of these studies were different compared to our work as they did not include patients with high myopia, and they did not use optical coherence tomography (OCT) for the detection of hypotony maculopathy. As there are no studies having as the first outcome the rate of hypotony and hypotony-related complications in patients with high myopia who underwent trabeculectomy, it is hard to compare our finding with published data. We used OCT as a routine postoperative examination in any patients who underwent glaucoma surgery. It is likely that the use of OCT has increased our diagnostic power as we were able to detect even subtle signs of hypotony maculopathy. Taking these considerations together, we can speculate that the reported rate of hypotony maculopathy up to 20% in non-highly myopic eyes can be compared with our rate of 42% of hypotony maculopathy detected by a high-resolution imaging technique in a population at high risk of hypotony-related complications.

The Xen 45 implant is a less invasive glaucoma surgical technique, leading to a filtering bleb by an ab interno approach. This device was designed to prevent hypotony, as the length/lumen ratio theoretically controls the aqueous outflow, according to the Hagen–Poiseuille equation [31]. Xen 45 and trabeculectomy have been compared in 12- and 24-month retrospective studies. Overall, the Xen 45 showed a safer postoperative profile compared to trabeculectomy. The incidence of hypotony ranged from 0% to 2.4% after Xen 45 and from 6.7% to 10.5% after trabeculectomy. In the retrospective study by Wagner and coauthors involving 82 cases of XEN implant and 89 cases of trabeculectomy, hypotony was reported in 6.7% of patients in the trabeculectomy and 2.4% of patients in the Xen group [32]. In a 24-month retrospective study, patients in the Xen 45 group experienced a significantly lower incidence of wound leak (0% vs. 15.8%), bleb leak (0% vs. 6%), and hypotony (1.8% vs. 10.5%) compared to trabeculectomy [17], and a retrospective study comparing trabeculectomy with Xen implant, both in combination with phacoemulsification, reported a higher incidence of hypotony after trabeculectomy (7.7% vs. 0%) [33]. In two retrospective Xen vs. trabeculectomy studies, the incidence of hypotony was not reported; however, the anterior chamber flattening, a condition commonly related to hypotony, was greatly more frequent in the trabeculectomy compared to the Xen group (19.6% vs. 1.5% and 14.7% vs. 0%, trabeculectomy vs. Xen, respectively) [34,35]. In one study, no late hypotony and hypotony maculopathy occurred after the first month of follow-up [36], and one study reported a similar incidence of choroidal folds and hypotony maculopathy in the two groups (hypotony maculopathy 1.1% Xen, trabe 0.6%) [16]. In a large, comparative retrospective study between standalone Xen 45 and standalone trabeculectomy [16], the authors reported a significantly lower percentage of patients with a vision loss of >2 lines in the Xen group.

The second outcome of our analysis was to report the efficacy of the two techniques.

Xen implant and trabeculectomy achieved a significant IOP reduction at 2 years of follow-up with a comparable survival curve. Although the IOP lowering efficacy was superior in the trabeculectomy group, we did not find a statistically significant difference at 1 and 2 years between the two techniques. The lower mean IOP we observed during the first 6 months in the trabeculectomy group is likely correlated with the higher proportion of hypotony that occurred in the trabeculectomy group. The mean IOP in the Xen 45 group was 12.43 and 15.0 at 1- and 2-year follow-ups. We found 28% and 7% of cases needing a needling procedure and a surgical revision, respectively. In large retrospective multicenter [37,38] and single-center [39,40] European studies, the mean IOP ranged from 14.6 to 15.5 and from 14.2 to 14.8 throughout the first and the second year of follow-up. The proportion of patients who underwent needling procedures ranged from 36% to 62% [37,38,39,40]. As none of these studies reported sub-analysis about patients with myopia or high myopia, it is hard to compare our results with existing literature.

We recently published a multicenter, retrospective study reporting the effectiveness and safety of Xen 45 in eyes with open-angle glaucoma (OAG) and high myopia [21]. Overall, the study by Fea et al. and the current study reported a significant reduction of IOP compared to baseline and a good safety profile in patients with glaucoma and high myopia who received a Xen implant. The IOP achieved at 1 year of follow-up and the rate of hypotony was similar in the two studies (12.6 mmHg vs. 12.0 mmHg, and 28.6% vs. 28.5%, Fea et al. and the current study, respectively).

Compared to the current study, the study by Fea and colleagues is multicentric and has a larger sample size but lacked a control group.

The baseline characteristics were similar between the studies in terms of mean deviation and number of antiglaucoma drugs (11.8 dB vs.10.88 dB, and 3.0 vs. 3.14, Fea et al. [21] and the current study, respectively), but patients in the Fea et al. study were older and had a higher baseline IOP, as well as a lower mean refraction (62.1 years vs. 52.9 years, 24.5 mmHg vs. 22.1 mmHg, −13.2 D vs. −15.07, Fea et al. [21] and the current study, respectively).

We are aware of the limitations of our study. First, the design of our work carries the limitations of any retrospective analysis.

The risk of selection bias due to the lack of randomization is counterbalanced by the lack of significant differences in the baseline characteristics of the two groups. As in any retrospective work, the rate of complications, specifically choroidal detachment and hypotony maculopathy, could be underestimated. However, this bias involves both groups equally. In addition, the threshold of attention in patients with high myopia is exceptionally high. Patients underwent a dilated funduscopic examination at any postoperative visit and an optical coherence tomography retina exam in the case of low visual acuity recovery and complaint of visual changes. Among the limitations, we are aware that the small sample size reduces the power of the study. However, our preliminary work aimed to explore the safety and efficacy of the Xen 45 implant in patients with glaucoma and high myopia, a relatively uncommon condition. As expected, the refractive characteristics we used as inclusion criteria of our analysis narrowed the sample size.

We also want to point out to be cautious in generalizing the results of this study and that the interpretation of our findings should be restricted to patients with similar baseline characteristics and in a similar setting.

Among the strengths, we defined strict inclusion and exclusion criteria using the hospital’s electronic database, and a single surgeon treated all the patients, thus minimizing the bias related to the surgical technique. We extended the follow-up to 2 years and included patients with the same refractive characteristics (high myopia) treated by trabeculectomy as a control group.

In conclusion, we report for the first time the safety and efficacy of Xen 45 implant in comparison with trabeculectomy in patients with concomitant glaucoma and high myopia. Myopic eyes are at higher risk of hypotony maculopathy after glaucoma surgery due to the sclera’s weakened biomechanical properties, including scleral thinning and reduced scleral stiffness. Xen implant showed a favorable safety profile, with a lower incidence of postoperative hypotony maculopathy compared to patients with similar characteristics treated with trabeculectomy. The safety results of our study after the Xen 45 implant agree with the available literature. Although the retrospective nature of our work and the limited sample size, we suggest considering Xen 45 implant in patients with high myopia requiring glaucoma surgery.

We hope this explorative work can draw attention to the management of patients with concomitant glaucoma and high myopia and will encourage further, larger works investigating this topic.

## Figures and Tables

**Table 1:** Preoperative data.

	Trabeculectomy (n = 7)	XEN Gel Stent (n = 7)	*p* Value *
Age (years)	63.9 ± 13.06	52.96 ± 10.15	0.1252
Sex (M/F), n (%)	1/6 (14.29/85.71%)	3/4 (42.86/57.14%)	0.5594 ^
Diabetes status (yes), n	1/7	1/7	---
Refraction (Sphere, D)	−14.71 ± 5.36	−15.07 ± 6.11	0.4776
Axial length (mm)	30.28 ± 2.38	30.85 ± 3.00	0.2225
BCVA (logMAR)	0.19 ± 0.13	0.43 ± 0.36	0.5261
Pseudophakic, n	5/7	4/7	0.5770
Type of glaucoma, n *POAG*	7/7	7/7	---
Intraocular pressure (mmHg)	22.00 ± 1.52	22.14 ± 4.88	0.3592
Pachymetry (µm)	519.71 ± 38.57	535.71 ± 26.03	0.5649
Glaucoma medications (N)	3.57 ± 0.53	3.14 ± 0.38	0.1246
Mean deviation (dB)	−16.17 ± 5.68	−10.88 ± 5.59	0.1252

Data are presented as mean ± standard deviation or frequencies and percentages. * Non-parametric Wilcoxon test; ^ Fisher’s exact test.

**Table 2:** Postoperative IOP values.

IOP (mmHg)	Trabeculectomy (n = 7)	XEN Gel Stent (n = 7)	*p* Value
1 day after surgery	5.43 ± 2.30	7.00 ± 1.41	0.1185
1 week after surgery	5.43 ± 2.44	8.43 ± 2.44	0.0440 *
1 month after surgery	5.43 ± 2.51	10.71 ± 1.25	0.0042 *
3 months after surgery	8.00 ± 2.58	13.00 ± 3.61	0.0324 *
6 months after surgery	8.50 ± 2.26 ^	14.50 ± 2.35	0.0059 *
1 year after surgery	10.00 ± 2.83 ^	12.00 ± 1.90	0.2240
2 year after surgery	12.83 ± 4.62	14.83 ±3.97	0.8717
*p*-value	<0.0001 #	<0.0001 #	

IOP = intraocular pressure. * Significant *p*-value (non-parametric Wilcoxon test). *#* Compared to baseline. ^ Fisher’s exact test.

**Table 3:** Postoperative complications.

	Trabeculectomy (n = 7)	XEN Gel Stent (n = 7)	Total (n = 14)
Hypotony	6 (85.71%)	2 (28.57%)	8 (57.14%)
Hypotony maculopathy	3 (42.86%)	0	3 (21.43%)
Choroidal detachment	4 (57.14%)	1 (14.29%)	5 (35.71%)
Flat anterior chamber	2 (28.57%)	0	2 (14.29%)
Hyphema	0	1 (14.29%)	1 (7.14%)
Bleb leakage	1 (14.29%)	0	1 (7.14%)
Endophthalmitis	0	0	0

**Table 4:** Postoperative management.

	Trabeculectomy (n = 7)	XEN Gel Stent (n = 7)	Total (n = 14)
Bleb needling	2 (28.57%)	2 (28.57%)	4 (28.57%)
Intrableb autologous blood injection	3 (42.86%)	0	3 (21.43%)
Laser suture lysis	1 (14.29%)	0	1 (7.14%)
AC viscoelastic injection	2 (28.57%)	0	2 (14.29%)
Surgical bleb revision	1 (14.29%)	1 (14.29%)	2 (14.29%)

AC = anterior chamber.

## Data Availability

The data presented in this study are available on request from the corresponding author. The data are not publicly available due to privacy restrictions.
